# Evaluating a Periapical Lesion Detection CNN on a Clinically Representative CBCT Dataset—A Validation Study

**DOI:** 10.3390/jcm13010197

**Published:** 2023-12-29

**Authors:** Arnela Hadzic, Martin Urschler, Jan-Niclas Aaron Press, Regina Riedl, Petra Rugani, Darko Štern, Barbara Kirnbauer

**Affiliations:** 1 Institute for Medical Informatics, Statistics and Documentation, Medical University of Graz, 8036 Graz, Austria; arnela.hadzic@medunigraz.at (A.H.); regina.riedl@medunigraz.at (R.R.); 2 Division of Oral Surgery and Orthodontics, Medical University of Graz, 8010 Graz, Austriapetra.rugani@medunigraz.at (P.R.); barbara.kirnbauer@medunigraz.at (B.K.); 3 Institute of Computer Graphics and Vision, Graz University of Technology, 8010 Graz, Austria

**Keywords:** artificial intelligence, deep learning, digital imaging/radiology, inflammation, oral diagnosis, periapical lesions, image segmentation, convolutional neural network

## Abstract

The aim of this validation study was to comprehensively evaluate the performance and generalization capability of a deep learning-based periapical lesion detection algorithm on a clinically representative cone-beam computed tomography (CBCT) dataset and test for non-inferiority. The evaluation involved 195 CBCT images of adult upper and lower jaws, where sensitivity and specificity metrics were calculated for all teeth, stratified by jaw, and stratified by tooth type. Furthermore, each lesion was assigned a periapical index score based on its size to enable a score-based evaluation. Non-inferiority tests were conducted with proportions of 90% for sensitivity and 82% for specificity. The algorithm achieved an overall sensitivity of 86.7% and a specificity of 84.3%. The non-inferiority test indicated the rejection of the null hypothesis for specificity but not for sensitivity. However, when excluding lesions with a periapical index score of one (i.e., very small lesions), the sensitivity improved to 90.4%. Despite the challenges posed by the dataset, the algorithm demonstrated promising results. Nevertheless, further improvements are needed to enhance the algorithm’s robustness, particularly in detecting very small lesions and the handling of artifacts and outliers commonly encountered in real-world clinical scenarios.

## 1. Introduction

Artificial intelligence (AI) models have made remarkable advancements in various fields, with deep convolutional neural networks (CNNs) [1] emerging as a powerful subset of AI, especially for processing and analyzing images. These networks, inspired by the structure of the visual cortex in the human brain, show superior performance in tasks such as image classification, object detection, and image segmentation [2]. In the field of medical imaging, these networks have demonstrated promising capabilities in detecting and diagnosing various diseases such as breast cancer, heart disease, and brain tumors [3,4]. Their performance is often reported to be comparable to that of experienced professionals, significantly reducing the time required for diagnosis [5,6,7].

Recently, dental medicine has also started to benefit from such deep learning techniques [8]. Specifically, these techniques have been applied to panoramic radiographs and cone-beam computed tomography (CBCT) images with the aim of assisting clinicians in detecting and analyzing dental conditions and diseases in the maxillofacial region [9,10,11]. Examples include the detection of maxillary sinus mucosa [12], pharyngeal airway space [13], calcifications of the cervical carotid artery [14], jaw cysts [15,16], supernumerary mesio-buccal root canals on maxillary molars [17], vertical root fractures [18], and periapical lesions (PALs).

PALs are one of the most frequent pathological occurrences in dental images. They resemble usually bacteria-induced osteolytic areas around the tip of the roots within a few millimeters in diameter [19,20]. PALs are conventionally analyzed in radiographs, whereas CBCT images often reveal these lesions as incidental findings [20,21]. While widely used conventional intra- and extra-oral radiographs [22] lead to lower radiation doses but suffer from superimposition issues due to their projective nature, CBCT allows fully three-dimensional (3D) imaging of the maxillofacial region at the cost of higher dose requirements. However, due to its volumetric nature, CBCT has been shown to improve the detection of PALs when compared with radiographs [23,24,25]. Manually identifying PALs with high sensitivity (recall) in both imaging modalities requires a certain amount of experience to prevent overlooked findings. As a result, automated deep learning-based methods for PAL detection in radiographs or CBCT imaging data have been proposed [16,26,27,28,29,30,31,32,33,34]. Serving as the foundation of this study, the promising CNN-based approach for periapical lesion detection in CBCT images proposed in [32] achieved a sensitivity of 97.1% and a specificity of 88.0% when evaluated on 144 CBCT volumes with 206 lesions.

The great success of any deep CNN-based approach is based upon the assumption that training and testing data come from the same data distribution. However, when the test data deviates from the training data distribution, the ability of deep neural networks to generalize and perform well on the new data degrades [35]. This phenomenon is often observed in clinical datasets due to factors such as anatomical anomalies, image artifacts, or occlusions, which shift the data distribution. In light of this, our validation study aims to provide a thorough statistical evaluation regarding the effectiveness and generalization capability of the CNN-based PAL-detection model proposed in [32] on an entirely new, previously unseen clinical CBCT dataset with a shifted data distribution compared to the data used to train the model. The null hypothesis of this validation study is that the method proposed in [32] delivers an inferior result when applied to our new, challenging evaluation dataset from clinical practice.

## 2. Materials and Methods

### 2.1. Study Design

The research protocol for this retrospective study was performed following the guidelines of the Declaration of Helsinki. Ethical approval for retrospective collection of the evaluation dataset used in this study was provided by the local Ethics Committee of the Medical University of Graz, Austria (review board number “34-519 ex 21/22”).

### 2.2. Sample Size Calculation

The sample size for this study is based on assumptions for the lesion detection sensitivity. We assumed observation of a sensitivity of 95%, corresponding to the lower limit of the 95% confidence interval for the sensitivity observed in the prior study of Kirnbauer et al. [32]. To show that the sensitivity is non-inferior to 90% (using a margin of 5%), a sample size of 243 lesions is necessary to achieve a power of >80%, using a one-sided non-inferiority binomial test with an alpha of 2.5%. Assuming that about 10% of the teeth have lesions, a sample size of 2430 teeth is required. For an assumed specificity of 87% and to show that the specificity is non-inferior to 82% (margin of 5%), the sample size of 2187 teeth yields a power of >99%.

### 2.3. Dataset

Dataset collection was performed similarly to Kirnbauer et al. [32], but in a less selective manner, so that clinical practice was better reflected (see the comparison of inclusion and exclusion criteria between studies in Table 1). CBCT volumes from routine clinical operations performed for different diagnostic indications (i.e., implant planning, radiological assessment of impacted teeth, assessment of odontogenic tumors or other lesions, and orthodontic reasons) from the year 2018 were retrospectively screened and selected according to the criteria listed in Table 1. All scans were performed on a Planmeca ProMax^®^ 3D Max (Planmeca, Helsinki, Finland) device.

The collected dataset was pseudonymized, so that patient names were replaced with a sequential code and no conclusions could be drawn about patient data when they were used during the investigation. All investigators who received access to encrypted and non-encrypted data were subject to the General Data Protection Regulation (GDPR) and the Austrian Data Protection Regulation in the currently valid version (http://www.dsb.gv.at, accessed on 14 December 2023). An initial dataset screening was performed by one dentist on an MDNC-2221 monitor (resolution 1600 × 1200; size 432 mm × 324 mm; 59.9 Hz; Barco Control Rooms GmbH, Karlsruhe, Germany) using the Planmeca Romexis^®^ software version 6.0 (Planmeca, Helsinki, Finland).

The ground-truth detection of PALs was performed by three investigators (two senior oral surgeons with >15 years of experience and one junior dentist), who did an initial round of lesion detection separately from each other on the whole dataset. Within a second round, lesion results were consensually determined including PAL classification, according to the periapical index scoring scheme of Estrela et al. [20], thus establishing the expert ground truth. The collected dataset consisted of a total of 196 CBCT images from unique patients. One patient image had to be excluded due to the software failing to read the file, leaving 195 patient images (99.5%) for the comparison of software-based PAL detections with expert ground truth within this study. Out of these 195 images, 164 showed only one jaw, and 31 images displayed both jaws. In total, there were 2947 present teeth across the 226 jaws (101 lower jaws, 125 upper jaws) in the dataset. In the images of these jaws, there were 669 teeth missing due to various possible reasons, such as caries, periodontitis, dental trauma, periapical disease, or orthodontic reasons [36].

During the investigation, a total of 300 periapical lesions were identified by the expert consensus, and the remaining 2647 present teeth were determined to be lesion-free. Table 2 provides a summary of the dataset characteristics, including lesion classification according to the lesion diameter-based periapical index scoring scheme proposed by Estrela et al. [20], while Table 3 gives a detailed distribution of tooth groups per lesion class.

### 2.4. Automatic PAL Detection

To facilitate the evaluation of the CNNs’ performances on the newly collected dataset, we have developed a user-friendly Windows software program. The program incorporates the PAL-detection method proposed in [32] and includes a graphical user interface built using the tkinter library in Python. This interface eliminates the need for any programming operations, thus simplifying the evaluation process and hiding the details of the software for the purpose of this independent evaluation. The user can select the CBCT image to be processed and choose the specific jaw they want to investigate. The software then creates a segmentation map of the detected lesions in the CBCT input image and saves it automatically. The time required to generate a segmentation map depends on the GPU used. Here, we utilized the Asus GeForce^®^ GTX 1660 Ti 6GB TUF Gaming EVO OC graphics card, and the generation of a segmentation map took at most 3 min.

**Figure 1:**
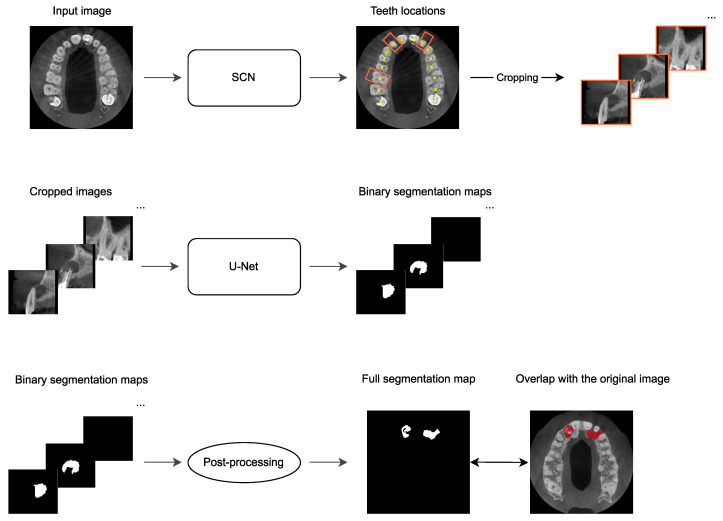
Method overview: First, the SpatialConfiguration-Net (SCN) is utilized to generate the locations of teeth in a given CBCT image. These locations are then used to crop the input image for each tooth, ensuring that the center coordinate of each cropped image matches the predicted coordinate of the corresponding tooth. In the second stage, the cropped images are fed into a modified U-Net, which generates binary segmentation maps that visualize detected lesions in these cropped images. Lastly, the binary segmentation maps are resampled and merged to create a full segmentation map that visualizes all detected lesions in the input CBCT image.

The PAL-detection method [32] integrated into the software consists of three main steps, as illustrated in Figure 1. First, the SpatialConfiguration-Net (SCN) [37] is trained to predict the 3D coordinates of teeth in the original images at a lower resolution. The original image is resized to a fixed aspect ratio of [64, 64, 32] before being input into the SCN. The resulting teeth locations are then utilized to crop the input image for each present tooth, where the center coordinates of the cropped images correspond to the predicted coordinates of teeth. Each cropped image is resampled using trilinear interpolation and an isotropic voxel size of 0.4 mm and has a size of [64, 64, 64]. Finally, these cropped images are fed into a modified U-Net, trained to generate binary segmentation maps that visualize the detected periapical lesions for individual teeth. The SCN and the U-Net were trained and tested on a dataset consisting of 144 CBCT images, with 2128 present teeth and 206 manually annotated periapical lesions. Within that dataset, there were 54 images of the lower jaw, 74 images of the upper jaw, and 16 images of both jaws. The method underwent a four-fold cross-validation, where the dataset was divided into four subsets of equal sizes, and the teeth with lesions were uniformly distributed over the folds. Each fold involved training on the three subsets and testing on the single remaining subset. As a result, four different trained models were obtained, with each model being trained on 108 images and tested on 36 different images. The models were trained in such a manner that the imbalance between positive and negative samples, i.e., cases with and without lesions, did not affect their performances. The training and testing procedures were performed using an Intel(R) CPU 920 with an NVIDIA GeForce GTX TITAN X on the Ubuntu 20.04 operating system with Python 3.7 and TensorFlow 1.15.0. The SCN required approximately 20 h for training per cross-validation fold, while the modified U-Net took approximately 17 h. For additional details on the network’s architecture, training/testing procedure, and prediction performance, we refer to [32,38].

The software used in this study utilizes one of the four pre-trained models from [32] to evaluate the lesion detection performance of the model on a new, independent dataset consisting of 195 CBCT images. The dataset used in this study is a pure test dataset that the model has not seen before, i.e., its images have not been used during training of the PAL-detection method or to tune its hyperparameters. Before generating the final output image, the software applies an additional post-processing step, as illustrated in the bottom row of Figure 1. After the PAL-detection method generates binary segmentation maps for the cropped teeth images, we implement a resampling and merging procedure to create a full segmentation map that includes detected lesions for all teeth within the original image. To achieve this, first, a blank segmentation map of the identical size and spacing as the original image is generated. Subsequently, the original input image and the blank segmentation map are resampled to match the spacing of the cropped images. The predicted teeth coordinates obtained from the SCN, which were used for cropping the images, are also transformed using the spacing of the cropped images. These transformed coordinates determine the regions where the predicted binary segmentation maps are copied. Finally, the full segmentation map containing the detected lesions is resampled back to match the size and spacing of the original image.

### 2.5. Expert Assessment of Software PAL Detections

After applying the automatic PAL-detection software (based on the pre-trained model from [32]) to the new dataset, lesion segmentation results were assessed in consensus by two of the three dental investigators (one senior, one junior), who first did the expert ground-truth annotation. For assessment, they used the ITK-SNAP software [39] and loaded the CBCT volume as well as the corresponding resampled lesion segmentation for visualization. Investigators marked the segmentation results, which were defined as regions of at least one voxel in size, as true or false positives on the level of individual teeth. As soon as the segmentation affected two or more neighboring teeth, all of those teeth were noted to have a CNN-detected PAL. Furthermore, segmentations that were lying in areas far away from the periapical regions, within nerve channels, pulp chambers of impacted teeth, or even outside the alveolar crest were also documented and detected as false positives.

### 2.6. Statistical Analysis

To evaluate the algorithm’s performance in identifying PALs, sensitivity and specificity metrics are used, comparing positive and negative detections with the expert ground truth. Sensitivity (recall) measures the method’s accuracy in identifying the presence of lesions, while specificity measures its ability to correctly identify the absence of lesions. Sensitivity and specificity with their corresponding exact 95% confidence intervals (CIs) are presented for all teeth and stratified by upper/lower jaw and tooth type. Non-inferiority tests were performed using one-sided binomial tests with an alpha of 2.5%. Differences in sensitivity and specificity between lower and upper jaws were assessed by Fisher’s exact tests. For statistical analysis, SAS version 9.4 was used.

## 3. Results

From Table 4, it can be seen that the overall sensitivity of the deep learning-based lesion detection approach evaluated on all present teeth was 86.7% (95% CI: 82.3–90.3%) when compared with the expert-derived ground truth. The specificity of the software was 84.3% (95% CI: 82.8–85.6%). The null hypothesis of inferiority of the software with respect to sensitivity could not be rejected (p=0.975), while the null hypothesis of inferiority with respect to specificity could be rejected (p=0.001). In our dataset consisting of images, where either one or both jaws were available, any of the jaws could have missing teeth. Out of a total of 669 missing teeth, the software found 42 false positive (6.3%) lesion predictions, while 627 missing teeth were correctly identified as negatives (93.7%). The confusion matrices of the overall results for present teeth, as well as for present and missing teeth combined, are given in Table 5 and Table 6.

Table 4 also illustrates individual lesion detection results stratified per jaw. For the upper jaw (N=125 patients with a total of 1598 present teeth), the sensitivity is 87.8% (95% CI: 82.3–92.0%), and the specificity is 82.3% (95% CI: 80.2–84.3%). For the lower jaw (N=101 patients with a total of 1349 present teeth), the sensitivity is 84.6% (95% CI: 76.2–90.9%), and the specificity is 86.4% (95% CI: 84.4–88.3%). The difference in sensitivity between the upper jaw and lower jaw is 3.2% (95% CI: −5.2–11.5%). This difference is not significant according to Fisher’s exact test when comparing the two jaws (p=0.478). The difference in specificity between the upper jaw and lower jaw is −4.1% (95% CI: −6.9–1.4%), which is significant (p=0.004) according to Fisher’s exact test.

Moreover, we illustrate lesion detection results stratified per tooth type (combined for both jaws) in Table 4. Sensitivities are below 70% for the three categories where also the total number of lesions was comparatively lower (third molars, canines, lateral incisors). On the other hand, for the remaining five tooth categories, the average sensitivity is 88.9%.

**Figure 2:**
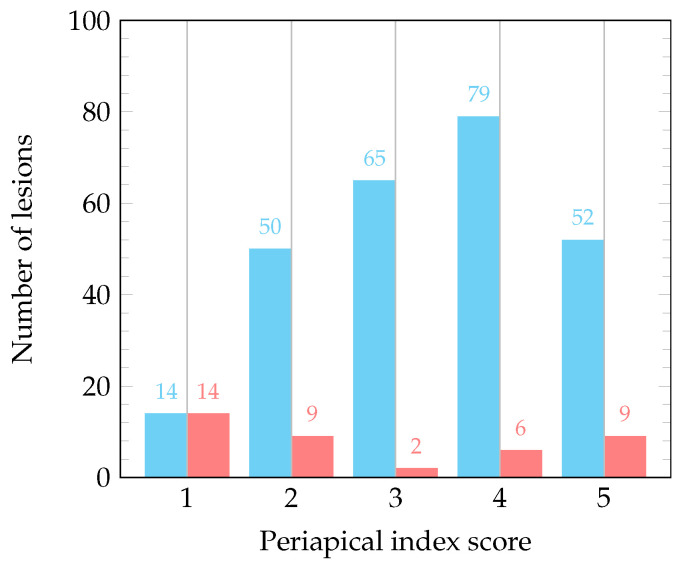
Distribution of predicted lesions across different periapical index scores according to Estrela et al. [20]. Blue bins represent true positive (TP) predictions, while red bins represent false negative (FN) predictions for a specific periapical index score.

Finally, we analyze the lesion detection results with respect to lesion classifications (periapical index scores according to Estrela et al. [20]). In Figure 2, we plot a histogram of true positives and false negatives per lesion type, illustrating that for the smallest lesion type (class 1, with a diameter of periapical radiolucency between 0.5 and 1 mm), the sensitivity is low, while for classes 2 through 5 (diameters of periapical radiolucency larger than 1 mm, see also Table 2), the sensitivities are much higher.

**Figure 3:**
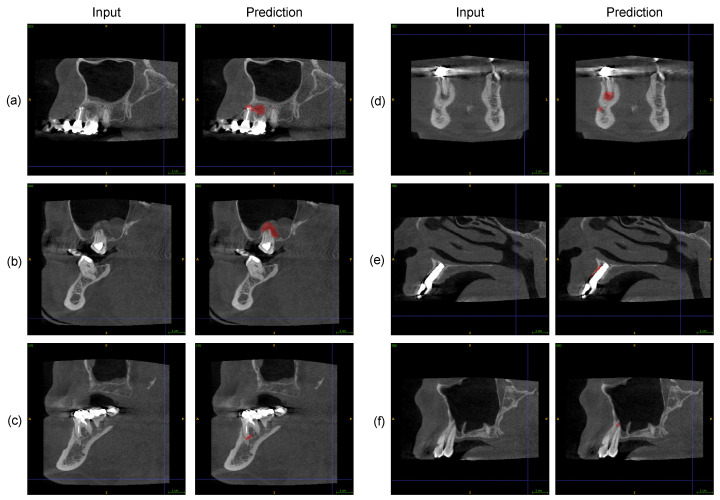
Qualitative results: (**a**) Two true positive (TP) lesions are detected at the second premolar and the first molar, and one false positive (FP) lesion is identified at the second molar in the upper jaw. (**b**) One TP lesion is found at the second molar in the upper jaw at a challenging location close to the sinus. (**c**) One TP lesion is detected at the first molar in the lower jaw. (**d**) One TP lesion is identified at the second premolar, along with one FP lesion at the mental nerve in the lower jaw. (**e**) One FP lesion is observed at the anterior tooth position in the upper jaw due to artifacts at an implant site. (**f**) One FP lesion is detected at the first premolar in the upper jaw (close to the sinus). The qualitative results are best visualized in PDF format.

Exemplary qualitative results of the software are shown in Figure 3.

## 4. Discussion

Recent machine learning methods, especially deep neural networks for assisting experts in the detection and segmentation of lesions in medical imaging data, have shown tremendous success, but they struggle with issues due to a lack of generalization to datasets from clinical practice [35]. We have performed a thorough evaluation and non-inferiority testing of a recently published algorithm for automated periapical lesion segmentation from dental CBCT images [32]. This algorithm was hidden behind a graphical user interface that solely produced a lesion segmentation given an input image from the new, single-use testing dataset used in this study. The dataset comprises 196 subjects with images of adult upper and lower jaws, including tooth gaps, dental restorations, implants, and impacted third molars (see Table 1). Additionally, the new dataset was obtained with a specific criterion that allowed the inclusion of up to 11 missing teeth per jaw. This led to a significantly higher number of missing teeth compared to the dataset in [32], where the aim was to include jaws with a minimal number of missing teeth. Thus, our new evaluation dataset reflects the presence of challenging circumstances in clinical practice. Moreover, our evaluation protocol was very strict in defining false positive findings, since one false positive (FP) voxel in the segmentation had already led to an FP prediction (see Figure 3a), thus imposing a hard but realistic scenario for the algorithm.

The algorithm could be successfully applied to present and missing teeth from 195 subjects, i.e., 99.5% of subjects in the total dataset. Our main result is a sensitivity of 86.7% and a specificity of 84.3% in detecting PALs at present teeth. The non-inferiority tests, which were designed upon sensitivity and specificity estimates derived from the proof of concept evaluation in [32], provided enough evidence to reject the null hypothesis for specificity but did not do so for sensitivity. Despite this drop in sensitivity, we still consider our absolute performance on this challenging dataset as very promising (see also our qualitative results in Figure 3a–c), since both the sensitivity and specificity are better than the threshold of 80%, which, according to the systematic review in [24], can be interpreted as the threshold indicating excellent results. One of the reasons for false positives occurring might be that some lesions are located close to the incisive, the inferior alveolar, and the mental nerve, as illustrated in Figure 3d. Furthermore, artifacts in general, caused by root canal fillings or dental implants potentially pose problems for the deep CNN (see Figure 3e). We also studied the algorithm’s performance at missing teeth and found that the overall specificity for present and missing teeth combined increases from 84.3% to 86.2% (see confusion matrix in Table 6).

Regarding the related work for automated detection of PALs in CBCT images, only a limited number of studies have been published. Zheng et al. [28] proposed an anatomically constrained Dense U-Net model, which they evaluated on 20 CBCT images, obtaining a sensitivity of 84.0% and a precision of 90.0% in a root-based evaluation. In addition, Orhan et al. [29] used a U-Net-based model to evaluate PAL detection in CBCT images and achieved a sensitivity of 92.8%. Setzer et al. [27] evaluated a U-Net-based model on 2D slices from 20 CBCT images and achieved a sensitivity of 93.0% and a specificity of 88.0% in PAL detection. Recently, Calazans et al. [34] proposed a classification model based on a 2D Siamese Network combined with a DenseNet-121 CNN [40]. Their model was evaluated on 1000 coronal and sagittal slices extracted from CBCT images and achieved a sensitivity of 64.5% and a specificity of 75.8% in classifying PALs.

Comparing our study with those conducted by Zheng et al. [28] and Orhan et al. [29] is difficult due to the lack of reported specificities and details regarding negative class examples in their research. Relying on the precision metric for comparison may be misleading since our dataset is highly imbalanced, whereas their datasets have a well-balanced distribution that does not reflect real-world clinical scenarios. The precision metric is sensitive to class distribution, making it less suitable in this context. In terms of sensitivity and specificity, our study outperforms the results of Calazans et al. [34], as they report a higher number of false negatives and false positives. While our sensitivity and specificity results are lower than those of the closely related and best-performing work by Setzer et al. [27], it is important to note that their evaluation solely consisted of 20 CBCT test images with 61 roots. Therefore, we claim that our evaluation protocol is more strict than theirs, due to our extensive single-use testing dataset collected from clinical practice. Furthermore, many of these works use models trained on 2D slices, thus neglecting valuable 3D information.

In CBCT imaging, PALs are often not the primary clinical question, however, secondary PAL findings occur frequently, and, furthermore, they have to be documented by dentists who are often not radiological experts or may not have sufficient time to assess the CBCT images in great detail. In such cases, the help of an algorithm is invaluable to prevent findings from being overlooked, even at the cost of a larger number of false positives, which, however, can be ruled out comparatively straightforwardly, either visually or via additional clinical assessment of the respective tooth.

To study our evaluation results in more detail, we also analyze different stratifications of the testing dataset. While collecting the expert ground truth of the lesions, a lesion classification of lesion diameters into five different periapical index score categories [20] was used. We see from Figure 2 that for lesion classes 2 through 5 (lesions with diameters larger than 1 mm), the algorithm leads to few false negatives, i.e., high recall, while for lesion class 1 (lesions between 0.5 and 1 mm of diameter), 50% of the lesions in our dataset were missed. From a radiological point of view, such small lesions are generally challenging to detect, which was previously reported by Tsai et al. [41] when studying simulated lesions in vitro on radiographs and CBCTs. If we use the lesion class stratification to compute the sensitivity solely for lesion classes 2 through 5, it reaches 90.4% (95% CI: 86.3–93.7%), which we consider to be a meaningful recall in clinical practice, such that the use of the algorithm can be suggested for lesions of sizes larger than 1 mm.

Another stratification that we investigated was from the anatomical point of view. Our results indicate that the algorithm provides a significantly higher specificity for teeth in the lower jaw, while the sensitivity difference between the lower and upper jaw was not statistically significant. We assume that this decrease in false positive findings for the lower jaw is due to its better radiological assessability compared with the upper jaw since the contrast between radiolucent lesions and alveolar bone or teeth is higher in the lower jaw (see Figure 3a (at the second molar), c and d). Moreover, teeth in the upper jaw are located close to the maxillary sinus, such that the thin bony maxillary sinus floor or potential sinus membrane alterations might lead to confusion for the algorithm (see Figure 3b,f).

When looking at different tooth categories in Table 4, where teeth are assessed for both jaws combined, we notice that there are three tooth groups for which sensitivity is lower (below 70%), i.e., wisdom teeth (3rd molars), canines, and lateral incisors. Wisdom teeth are rarer in the population in general since many of them are removed when reaching adulthood or never show up. This is also reflected in our dataset, thus leading to a low number of lesions as well (see Table 4). Moreover, different from lateral incisors and canines, molars are the teeth most affected by PALs, according to [42]. Due to the lower number of lesions in the abovementioned three tooth groups (see Table 4 for numbers of lesions), false negatives have a larger relative influence. Additionally, class 1 lesions of a smaller diameter are more strongly represented in two out of these three tooth categories in our dataset (third molars, lateral incisors, see Table 3). We assume that the combination of these aspects leads to the lower sensitivity, while the average sensitivity of the remaining five tooth categories, where a larger number of lesions is present in each category, is 88.9%.

One limitation of our study is that the dataset collection for this evaluation was performed at the same hospital as in [32]. While the focus was on the evaluation of generalizability via the inclusion of challenging data representative for clinical practice, we can therefore not draw any conclusions regarding generalizing to data from different sites. Moreover, the impact of anatomic variability due to differences in ethnicities, as, e.g., demonstrated in [43], regarding the occurrence of radix entomolaris in an Asian population, has not been taken into account. Another limitation was that our testing dataset only contained a low number of lesions of periapical lesion index score 1. We conclude that in order to improve the algorithm and to draw stronger conclusions for small lesions as well, a re-training of the machine learning method on more data with the class 1 lesion diameter is required, which we see as potential future work.

In summary, we see our results as a very promising indication that machine learning can play an important role in assisting experts in dental practice, where high recall is needed to prevent overlooked findings.

## 5. Conclusions

In this validation study, we performed a thorough evaluation and non-inferiority test of the periapical lesion (PAL)-detection algorithm proposed in [32] using a new, real-world clinical CBCT dataset. Despite the presence of challenging scenarios in the dataset, such as dental restorations, implants, and impacted third molars, the algorithm demonstrated promising results in accurately detecting PALs. Our evaluation covered all present teeth in the dataset and yielded a sensitivity of 86.7% and a specificity of 84.3% when compared with expert ground truth. The non-inferiority test rejected the null hypothesis for specificity for the non-inferiority threshold of 82%, but it did not reject the null hypothesis for sensitivity for the non-inferiority threshold of 90%. We also found that for lesions smaller than 1 mm, the sensitivity was low. However, when evaluating solely on lesions with periapical index scores 2 through 5, the sensitivity increased to 90.4%, thus indicating that the algorithm has the potential to assist clinicians to prevent overlooked lesions with a diameter above 1 mm. Overall, we conclude that the algorithm is promising but not yet fully robust to all the artifacts and outliers that were present in this clinically representative dataset.

## Figures and Tables

**Table 1:** Comparison of inclusion and exclusion criteria for dataset collection in this study and the study conducted by Kirnbauer et al. [32].

Criterion	Kirnbauer et al. [32]	This Study
Field of view with a representation of the entire dental arch (upper jaw, lower jaw, or both)	Included	Included
Device and assessment parameters: Field of view of 10.0 × 5.9 cm or 10.0 × 9.3 cm, covering at least one completely visible dental arch, with a 200-µm voxel size (96 kV, 5.6–9.0 mA, 12 s), which is labeled as “normal” mode by the manufacturer	Included	Included
An acceptable degree of scatter and/or artifacts (exclusion of clinically insufficient interpretable datasets, i.e., severe metal artifacts inhibiting individual crown visualization, and ghost effects/double images due to long-motion artifacts)	Included	Included
Completed root development	Included	Included
No edentulism	Included	Included
	Additional:	Additional:
	as few missing teeth as possible	up to 11 missing teeth per jaw
Tooth gaps	Excluded	Included
Partially and totally impacted teeth	Excluded	Included
Dental implants	Excluded	Included
Augmentations	Excluded	Included

**Table 2:** Overview of dataset characteristics used for the evaluation in this study.

	Number	Additional Information
Images	195	One jaw: 164
		Both jaws: 31
Jaws	226	Upper: 125
		Lower: 101
Teeth present	2947	With lesion: 300 (10.2%)
		Without lesion: 2647 (89.8%)
Lesion classification ^1^	Score 1: 28 ( 9.3%)	Diameter > 0.5–1 mm
	Score 2: 59 (19.7%)	Diameter > 1–2 mm
	Score 3: 67 (22.3%)	Diameter > 2–4 mm
	Score 4: 85 (28.3%)	Diameter > 4–8 mm
	Score 5: 61 (20.3%)	Diameter > 8 mm

^1^ Lesion classification performed according to Estrela et al. [20].

**Table 3:** Distribution of lesions over periapical index scores for each tooth group.

Periapical Index Score	1	2	3	4	5	Total
Third molars	3 (27.3%)	4	0	2	2	11
Second molars	4 ( 6.0%)	12	9	20	22	67
First molars	3 ( 3.5%)	17	19	25	21	85
Second premolars	6 (14.0%)	9	10	14	4	43
First premolars	2 ( 5.7%)	9	11	8	5	35
Canines	1 ( 7.7%)	3	4	2	3	13
Lateral incisors	3 (21.4%)	1	4	4	2	14
Central incisors	6 (18.8%)	4	10	10	2	32
Total	28 (9.3%)	59	67	85	61	300

**Table 4:** Lesion detection results. We show sensitivities and specificities including confidence intervals (CIs) for all present teeth in three result categories: overall for all teeth, stratified by jaws, and stratified by tooth type (combining jaws).

Category	Lesion count	Sensitivity (%)	95% CI Exact	Specificity (%)	95% CI Exact
Overall	300	86.67	82.29–90.30%	84.25	82.80–85.61%
Upper jaw	196	87.76	82.33–91.99%	82.31	80.21–84.27%
Lower jaw	104	84.62	76.22–90.94%	86.43	84.40–88.28%
Third molars	11	63.64	30.79–89.07%	81.61	75.04–87.07%
Second molars	67	91.04	81.52–96.64%	70.59	64.97–75.78%
First molars	85	91.76	83.77–96.62%	70.51	64.22–76.28%
Second premolars	43	88.37	74.92–96.11%	81.63	77.03–85.64%
First premolars	35	82.86	66.35–93.44%	87.37	83.60–90.54%
Canines	13	69.23	38.57–90.91%	89.70	86.41–92.41%
Lateral incisors	14	64.29	35.14–87.24%	92.68	89.72–95.01%
Central incisors	32	90.63	74.98–98.02%	88.03	84.44–91.04%

**Table 5:** Confusion matrix of lesion detection for present teeth.

		Predicted condition		
		Lesion	Non-lesion	Total
Actual condition	Lesion	260	40	300
	Non-lesion	417	2230	2647
	Total	677	2270	2947

**Table 6:** Confusion matrix of lesion detection for present and missing teeth combined.

		Predicted condition		
		Lesion	Non-lesion	Total
Actual condition	Lesion	260	40	300
	Non-lesion	459	2857	3316
	Total	719	2897	3616

## Data Availability

The data presented in this study are available on request from the corresponding author. The data are not publicly available due to ethical restrictions.

## Abbreviations

The following abbreviations are used in this manuscript:

CBCT	Cone-beam computed tomography
AI	Artificial intelligence
CNN	Convolutional neural network
3D	Three-dimensional
PAL	Periapical lesion
GDPR	General Data Protection Regulation
SCN	SpatialConfiguration-Net
CI	Confidence interval
TP	True positive
FN	False negative
FP	False positive
